# Vascular Leaking, a Pivotal and Early Pathogenetic Event in Systemic Sclerosis: Should the Door Be Closed?

**DOI:** 10.3389/fimmu.2018.02045

**Published:** 2018-09-07

**Authors:** Cosimo Bruni, Tracy Frech, Mirko Manetti, Francesca Wanda Rossi, Daniel E. Furst, Amato De Paulis, Felice Rivellese, Serena Guiducci, Marco Matucci-Cerinic, Silvia Bellando-Randone

**Affiliations:** ^1^Division of Rheumatology, Department of Experimental and Clinical Medicine, University of Florence, Florence, Italy; ^2^Division of Rheumatology, Department of Internal Medicine, Salt Lake Veterans Affair Medical Centre, University of Utah, Salt Lake City, UT, United States; ^3^Section of Anatomy and Histology, Department of Experimental and Clinical Medicine, University of Florence, Florence, Italy; ^4^Department of Translational Medical Sciences, Center for Basic and Clinical Immunology Research (CISI), WAO Center of Excellence, University Federico II, Naples, Italy; ^5^Division of Rheumatology, Department of Medicine, University of California, Los Angeles, Los Angeles, CA, United States; ^6^Department of Rheumatology, University of Washington, Seattle, WA, United States; ^7^Division of Rheumatology and Scleroderma Unit, Department of Geriatric Medicine, Azienda Ospedaliero Universitaria Careggi, Florence, Italy

**Keywords:** systemic sclerosis, edema, capillary leak, extravasation, vasculopathy, endothelial dysfunction, permeability

## Abstract

The early phase of systemic sclerosis (SSc) presents edema as one of the main features: this is clinically evident in the digital swelling (puffy fingers) as well as in the edematous skin infiltration of the early active diffuse subset. Other organs could be affected by this same disease process, such as the lung (with the appearance of ground glass opacities) and the heart (with edematous changes on cardiac magnetic resonance imaging). The genesis of tissue edema is tightly linked to pathological changes in the endothelium: various reports demonstrated the effect of transforming growth factor β, vascular endothelial growth factor and hypoxia-reperfusion damage with reactive oxygen species generation in altering vascular permeability and extravasation, in particular in SSc. This condition has an alteration in the glycocalyx thickness, reducing the protection of the vessel wall and causing non-fibrotic interstitial edema, a marker of vascular leak. Moreover, changes in the junctional adhesion molecule family and other adhesion molecules, such as ICAM and VCAM, are associated with an increased myeloid cells' extravasation in the skin and increased myofibroblasts transformation with further vascular leak and cellular migration. This mini-review examines current knowledge on determinants of vascular leak in SSc, shedding light on the role of vascular protection. This could enhance further studies in the light of drug development for early treatment, suggesting that the control of vascular leakage should be considered in the same way that vasodilation and inflammation reduction, as potential therapeutic targets.

Systemic sclerosis (SSc) is characterized, in its early phase, by the prominent interplay between the microvasculature and the immune system (1). The injury to the endothelium and the vessel wall, the activation and perivascular homing of inflammatory cells and the contemporary loss of the vascular tone control are a major triad contributing to the initiation and maintenance of vascular leak (2). The aim of this review is to examine the characteristics and mechanisms of vascular leaking in SSc.

## Anatomy

The vessel structure depends on its size and function: while larger arteries, arterioles, veins and venules have an endothelial layer plus varying amounts of surrounding muscular cells, capillaries and post-capillary venules have an inner surface coat overlying the endothelium called the glycocalyx, a negatively charged glycosaminoglycan (GAG) layer, and are usually surrounded by pericytes (3). The endothelium represents a barrier to extravasation, preventing circulating cells and macromolecules from crossing the lipid membrane. Endothelial cells (ECs) are anchored via integrins to a basement membrane (BM) which can be fenestrated or continuous. Central nervous system, connective tissue, heart and muscle have a continuous endothelium: ECs are linked together with tight intercellular junctions, adherent junction (VE-cadherin and catenin) and tight protein and glycoprotein junctions (occludins, claudins, and junctional adhesion molecules -JAMs- family members) controlling cell trafficking and protein and fluids passage (4).

In specific conditions, an intercellular vascular leak can be a physiological reparative event, such as during neovascularization following wound healing. This is consecutively characterized by BM degradation, pericyte detachment, endothelial thinning and increase in lumen size, mostly at a post-capillary venule level (5).

## Vascular mediators and physiological permeability

Independent of BM structure, various angiogenic and lymphangiogenic mediators derived by several inflammatory effector cells (such as mast cells, eosinophils, basophils, macrophages, etc.) can regulate physiological vascular permeability and extravasation, such as the vascular endothelial growth factor (VEGF) (6). VEGF isoforms signal through different members of the VEGF receptors family, which are expressed on several cells including ECs. VEGF is a mitogen and a vasodilator stimulating vascular permeability (7, 8), affecting perivascular pericytes and concomitantly increasing cellular migration (9). Moreover, VEGF-A induces VE-cadherin phosphorylation thus impairing endothelial barrier integrity and increasing vascular permeability (10). Angiopoietin (Ang) system represents a complementary pathway in the regulation of vascular endothelial barrier function (11). In humans, Ang1 and Ang2 are, respectively, a full agonist and a partial agonist of the Tie2 receptor on ECs: the former inhibits endothelial permeability, the latter induces it (12, 13).

In addition, transforming growth factor beta (TGFβ), a potent inhibitor of ECs proliferation and migration, induces pericyte differentiation, production of BM and induces VEGF inhibiting Ang1 expression in pericytes and fibroblasts. Therefore, TGFβ can exacerbate vascular leak in certain pathological conditions (14).

Capillary permeability may be significantly increased by hypoxia-reperfusion injury. In a pig-heart model, in fact, reperfusion caused damage to the glycocalyx, with increased serum levels of heparan-sulfate derived from GAGs shedding, influenced by oxygen-derived free radicals and xanthine-oxidoreductase activity (15).

## Pathological capillary leaking

Capillary leak may take place in several diseases due to local pathological conditions and the following are prototypes arising from different origins:

1. Anti-angiogenic and potential permeability inhibitors have been demonstrated in neoplasms (16),
2. Hypoxia-induced VEGF production can determine vascular leak and local edema in ischemic diseases such as stroke and myocardial infarction (17),
3. Pathological or mechanical endothelial stretch induces increased vascular permeability in pulmonary diseases including asthma (18), acute and ventilator-induced lung injury (19),
4. Hypoxia-induced VEGF production has been demonstrated in ocular conditions such as diabetic retinopathy or age-related macular degeneration (20).

Similarly, systemic inflammatory pathologies such as sepsis, pancreatitis and major traumas may induce a capillary leak. These are characterized by an increase in pro-inflammatory mediators (e.g., G-CSF, IL-6, IL-8, sTNFR1), followed by an increase of endothelial protective proteins, like Ang1, and of heparin binding proteins derived from glycocalyx damage (21, 22).

A distinct pathological entity called Systemic Capillary Leak Syndrome is characterized by arterial hypotension, hemoconcentration and low albumin levels with hypotensive shock and anasarca. In these patients, pro-inflammatory and endothelial mediators are significantly increased (23) and prompt treatment is required (24).

## Vascular leaking in SSc

In SSc, endothelial injury is a pivotal pathological event (25) which may have twofold explanations. Firstly, several unknown toxic stimuli (including ischemia-reperfusion) may induce a state of persistent endothelial activation resulting in apoptosis and detachment. Altered vasculature causes the direct activation of the alternative complement and coagulation cascades, leading to abnormal platelet activation (26), which amplifies and maintains vascular permeability and promotes the formation of intravascular fibrin deposits contributing to intimal proliferation, luminal narrowing and vessel obstruction (26). Secondly, there is an ineffective ability to respond to all these insults by promoting vascular repair (27).

In the pathogenetic progression, the vascular leak could be due to the modification of endothelial glycocalyx, vesiculo-vacuolar organelles, extracellular matrix (ECM), BM, intercellular junctions, cytoskeletal proteins and/or vascular pericytes. Using electron microscopy, SSc skin capillaries revealed intercellular gaps, vacuolization and destruction of ECs, reduplication of the basal lamina, perivascular cellular infiltrates and fibroblasts and pericytes with an enlarged rough endoplasmic reticulum accompanied by perivascular fibrosis (28–30).

In early SSc, vasculopathy is paralleled by an increased production of pro-angiogenic factors (e.g., VEGF, endothelin-1) (31, 32) and ECs defective response. These early events lead to vascular tone dysfunction, reduced capillary blood flow and chronic tissue hypoxia, further exacerbated by ECM accumulation and fibrosis (32). Biomarkers of vascular leak in SSc suggest intracellular signaling cascades impact on endothelial cytoskeletal and junctional proteins. The localization and function of junctional proteins and vesicular bodies can be significantly influenced by vasoactive substances, inflammatory mediators, and mechano-transduction. Damaged cells and inflammatory cells produce signaling mediators, such as histamine, TGFβ and VEGF, that can directly increase vascular permeability (2, 8, 33, 34). While perivascular cellular infiltrates and EC damage seemingly precede fibrosis (28), once hyperpermeability or leak occurs, the ECM is important for the propagation of fibrosis through its direct influence on fibroblast-myofibroblast transition and endothelial-to-mesenchymal transition (EndoMT) and the generation of profibrotic myofibroblasts (35).

Taken together, these events lead to a microcirculatory endothelial dysfunction, characterized by inflammatory immune cells surrounding microvessels (36), and to an organ damage, both independent from the rate of fibrosis (37). The role of microvascular endothelium is pivotal in triggering the activation of tissue cells, such as dendritic cells and macrophages, through the presentation of self-antigens, and myofibroblasts, through the release of TGFβ and other cytokines and growth factors (26).

## Immune cells and vascular leaking in SSc

Genetic factors are implicated in SSc development and are related to immunity and inflammation, thus suggesting a crucial role of the autoimmune dysregulation in all the phases of the disease (1), including vascular leak onset (26, 27, 36–43).

The response of the innate immune system against pathogens or non-specific damage is achieved through the activation of the so-called pattern recognition receptors, in particular the Toll-like receptors (TLRs). TLRs are evolutionary conserved receptors that, upon binding to their ligands, trigger the inflammatory response and induce several cellular changes (44). In addition to microbial antigens, TLRs can recognize endogenous molecules, contributing to the pathogenesis of many autoimmune diseases, including SSc (45, 46). The damage-associated molecular patterns (DAMPs), released from endogenous cells upon necrosis or tissue injury, generated by stressed cells, or resulting from mechanical or biochemical fragmentation of extracellular molecules, serve as an alarm “signal” for cells via TLRs (47). The mechanism regulating DAMPs release and interaction with the microenvironment is still poorly understood. The EC stress and injury trigger the inflammatory response mainly through the activation of NF-kB. Aberrant TLR signaling may be involved at the onset and during progression of SSc, contributing significantly to tissue inflammation and aberrant wound healing process (47).

**Figure 1:**
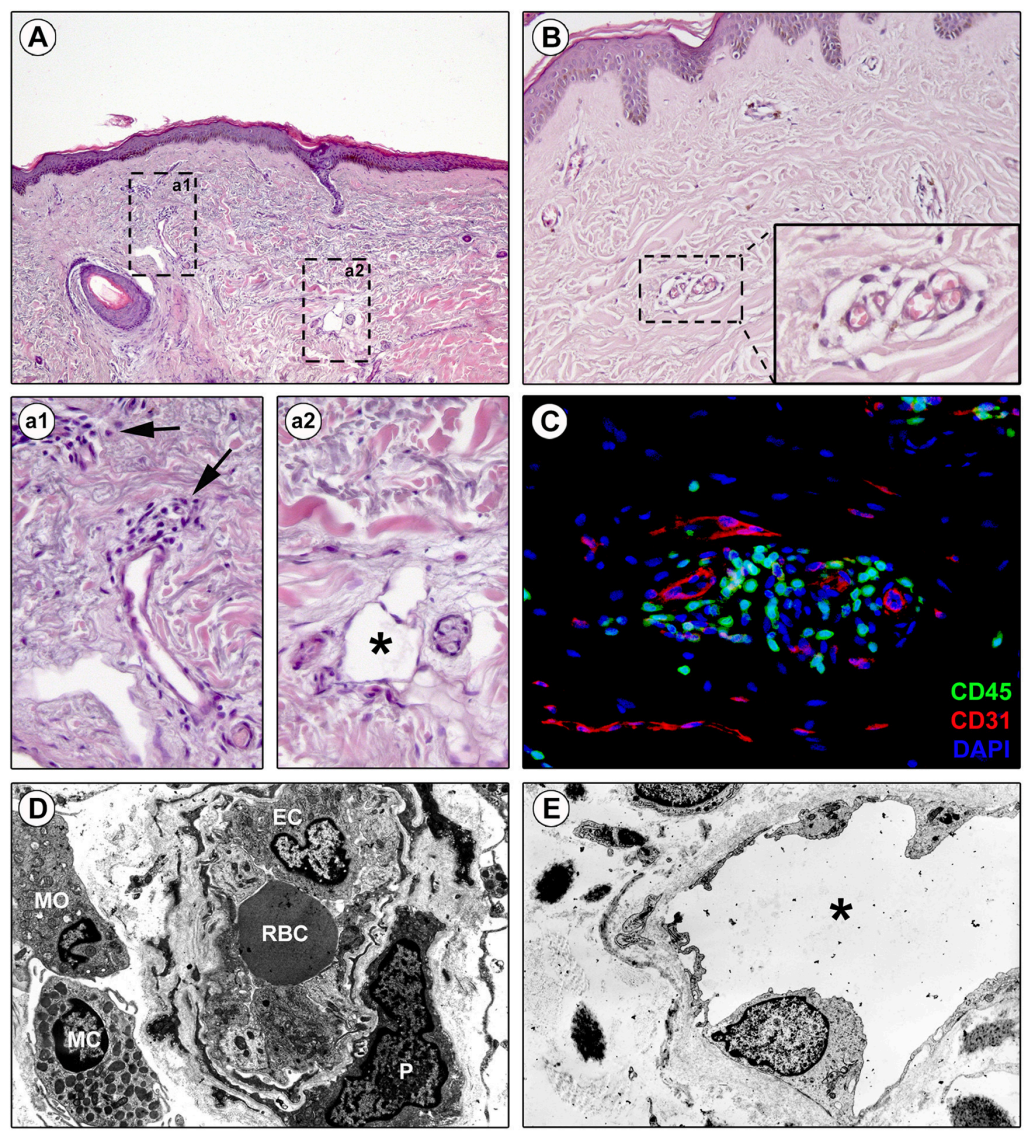
Perivascular edema and inflammatory cell infiltration are prominent in skin of early diffuse cutaneous systemic sclerosis (SSc). Representative microphotographs of paraffin-embedded skin sections from patients with early diffuse cutaneous SSc subjected to hematoxylin and eosin staining **(A, a1, a2, B)** or double immunofluorescence staining for CD45/leukocyte common antigen (green) and CD31/pan-endothelial cell marker (red) with 4′,6-diamidino-2-phenylindole (DAPI, blue) counterstain for nuclei **(C)** are shown. **(a1)** and **(a2)** represent higher magnifications of the boxed areas in **(A)**. **(a1)** Infiltrating inflammatory cells are observed around small dermal blood vessels (arrows). **(a2)** A dermal lymphatic vessel with an enlarged lumen (asterisk) is surrounded by edematous extracellular matrix. **(B)** Edema is prominent around blood capillaries. The inset depicts a higher magnification view of the boxed area from the respective panel. **(C)** CD45-positive inflammatory cells are widely found in the perivascular area. **(D,E)** Representative transmission electron microscopy microphotographs of ultrathin skin sections from patients with early diffuse cutaneous SSc. **(D)** A blood capillary displays hypertrophic endothelial cells and is surrounded by edema and inflammatory cells. **(E)** A lymphatic vessel surrounded by edema shows an enlarged lumen (asterisk). EC, endothelial cell; MC, mast cell; MO, mononuclear cell; P, pericyte; RBC, red blood cell.

Evidence suggests that the perivascular infiltrate participates in vessel wall remodeling (29). In early SSc, precursor cells such as monocytes, recruited from the bone marrow, migrate in the tissues (47) with T-cells and macrophages, to form the perivascular infiltrate between collagen fibers (Figures 1A,a1,C). Macrophages can generate different form of cells roughly categorized as classically activated (M1) and alternatively activated (M2) macrophages (48): M1 macrophages are effector phagocytes with an enhanced microbicidal or tumoricidal capacity, whereas M2-polarized macrophages are activated mostly by IL-4, IL-13, and IL-10, as well as by CCL2 and IL-6 (49). Commonly found during the peak of the profibrotic immune response, M2 macrophages have been proposed as inducers of wound healing, tissue fibrosis and remodeling in SSc. First, M2 macrophages suppress M1 responses and then promote collagen synthesis and profibrotic cytokine release, and Th2 effector responses. Consequently, if inflammation and tissue damage fail to resolve, a persistent fibrotic state may arise (50).

In the early edematous phase of SSc, the perivascular infiltrate is dominated by T-cells, with CD4^+^ T-cells predominating over CD8^+^ T-cells. The T-cell infiltrate is characterized by γδT-cells, mostly expressing the V1 chain (Vδ1^+^cells), which are a “non-conventional” T-cell population able to recognize non-peptidic antigens independent of major histocompatibility (MHC) molecule (51). These cells represent another potential mechanism that can contribute to the initial immune damage of SSc (52). The role of the γδT-cells is not completely understood. On one hand a defective regulatory function of γδT-cells may play a role in the breakdown of tolerance contributing to the early stages of the disease (53). Vδ1^+^-cells represent the majority of γδT-cells found in SSc skin lesions (54), peripheral circulation and bronchoalveolar lavage fluid suggesting an effector rather than regulatory function (55). In SSc, Vδ1^+^ γδT-cells are recruited by chemokines secreted by local cells (i.e., fibroblasts), starting immune-mediated endothelial damage. This is demonstrated by the presence of the activation marker CD49d mediating γδT-cells adherence to the ECs through the binding of the vascular cell adhesion molecule-1 (VCAM-1) (56). CD49d-VCAM-1 interactions are implicated in endothelial injury and cytotoxicity of activated γδT-cells (55).

In SSc vasculopathy evolution, the contact of leukocytes with ECs and fibroblasts is of paramount importance (57, 58). The expression and function of several cell adhesion molecules regulate the maintenance of trans-endothelial leukocyte migration. In affected SSc skin, specific EC activation markers are upregulated, including E-selectin, P-selectin, intercellular adhesion molecule (ICAM), JAMs, platelet EC adhesion molecule (PECAM), and vascular cell adhesion molecule (VCAM), particularly on ECs and fibroblasts in proximity to the perivascular infiltrate (55). Several other markers of EC activation, although expressed to a lesser extent, are also elevated in the sera of SSc or certain SSc subtypes (i.e., von Willebrand factor, fractalkine, β1/β2/β4 integrins, etc.) (58).

The complex role of the immune system in the pathogenesis of SSc is further highlighted by the detection of substantial differences in cytokine production in SSc, with differences according to cutaneous and internal organ involvement (59, 60).

Recently, the role of the adaptive immune system in SSc pathogenesis has been highlighted. In early SSc, Th1 and Th17 cells are predominant and pro-inflammatory cytokine release drives inflammation, whereas Th2 cells predominate in the later stages, participating through the release of profibrotic cytokines (60).

Mast cells (MCs) and basophils have also been implicated in the pathogenesis of systemic autoimmune diseases (SADs) (61, 62). It is becoming evident that MCs and basophils can be activated by a plethora of stimuli relevant in SADs (e.g., viral and bacterial products, complement, cytokines, etc.) and can also modulate innate and adaptive immune responses (18). MCs are implicated in different fibrotic conditions (renal fibrosis and pulmonary fibrosis) (60) and have also been identified in SSc dermis (47). Mast cells, due to their ability to inhibit Treg cells and enhance generation of Th17 cells, are potential antagonists of a proper development and function of Treg cells (1). Interestingly, peripheral blood basophils are upregulated in patients with SSc (63).

B-cells are yet another potential mediator of vascular injury. In fact, highly specific autoantibodies (anti-topoisomerase I, anticentromere and anti-RNA polymerase III antibodies) appear years before the clinical disease (64). In SSc, the immunoregulatory involvement of B-cells is demonstrated by the finding that anti-EC antibodies induce apoptosis. In SSc sera, a subset of autoantibodies against heterogeneous antigens on ECs (referred to as AECA) are able to induce microvascular EC apoptosis through antibody-dependent natural killer cell cytotoxicity (47).

Finally, the involvement of the immune system seems to be responsible not only for tissue inflammation but also as an inducer of fibroblast-myofibroblast transition (65).

## Cutaneous blood capillary leak as pathogenic initiator of lymphatic microangiopathy in SSc

Clinical and histological findings have shown that lesional SSc skin exhibits lymphatic microcirculation abnormalities that may be involved in the early edematous phase, when digital painless swelling is a clinical hallmark (66–68). Of note, early blood capillary leak may occur during the initiation of the cascade of lymphatic microangiopathy (66). Indeed, dermal blood capillary leaking causes greater amount of fluid and macromolecules in the interstitium, with maximal increase in lymph flow provoking micro-lymphatic insufficiency and consequent accumulation of protein-rich interstitial fluid, clinically manifest as edema (Figures 1A,a2,E) (66). The consequent inflammatory response and fibrotic process then perpetuate micro-lymphatic injury in a vicious circle similar to that occurring in chronic venous insufficiency. The investigation of the cutaneous capillary lymphatic system in SSc using fluorescence micro-lymphography revealed an augmented dye expansion into the superficial network of lymphatic capillaries and dermal backflow, postulated to be the consequence of hampered drainage of interstitial fluid into the deeper lymphatic collector vessels (66). Moreover, disease duration correlated with the loss of the micro-lymphatic network at the dorsum of the fingers (66). This evidence was further corroborated by histological studies reporting that the numbers of either lymphatic capillary or pre-collector vessels are decreased in SSc skin, with the reduction of the latter being more pronounced than that of capillaries (67, 68). In addition, such a loss of cutaneous micro-lymphatics appears closely linked to the progression of dermal fibrosis and development of fingertip ulcers (67–69).

## Clinical evidence and quantification of vascular leak in SSc

In SSc, capillaries not only undergo morphologic changes that suggest vascular leak (29), but also display functional deficits (70), resulting in a hyperpermeable state (Figures 1B–D). Recovery of the normal vasculature requires resolution of vascular leak (2). The inability of SSc microvessel to recover from vascular leak increases the edema from lymphatic microangiopathy (see above). In fact, the most typical SSc clinical presentation is vascular dysfunction, as manifested by Raynaud's phenomenon, followed by the onset of edematous puffy hands (71). As such, this early stage of hyperpermeability prior to the onset of fibrosis would be an ideal target for therapeutic intervention, thus highlighting the importance of its characterization. While the skin is the primary organ that has been studied in SSc, autopsy data suggests that all organs' microvasculature is affected (72). Indeed, vascular leaking can be also hypothesized in early lung involvement (with ground glass opacities on high resolution computed tomography) as well in the myocardial edema detected on cardiac magnetic resonance (73).

Non-invasive investigational studies support a progressive hyperpermeability of microvasculature in SSc. Nailfold videocapillaroscopy (NVC) is one of the major components in the 2013 classification of SSc (74), demonstrating progressive loss of the peripheral vascular network, dilatation of capillaries, deficient vascular repair and inadequate angiogenesis (75). While NVC is efficient for measuring morphological microvascular changes (microangiopathy), other methods, such as Laser Doppler, thermography or Near Infrared Spectrometry (76), are essential for the assessment of functional blood flow abnormalities. Results published so far do not clearly define the mechanistic changes of glycocalyx, ECM, BM, vesiculo-vacuolar organelles, cellular junctions, cytoskeletal proteins, and/or vascular pericytes abnormalities. Detailed longitudinal studies are in progress to define a unified vascular phenotype in SSc (77).

Evaluation of sublingual microcirculation can detect both morphological and functional capillary impairment and allows measurement of the glycocalyx layer. A pilot study found correlation of abnormal sublingual microcirculation, evaluated by intravital microscopy using Sidestream Dark Field (SDF) with a CapiScope HVCS handheld video capillary microscope (KK Technology, Honiton, UK), suggesting a decrease in glycocalyx thickness. Correlation between NVC capillary density and sublingual capillary density measured by SDF was discovered, supporting the concept of diffuse multi-organ microcirculation abnormalities in SSc (78). In another study of sublingual microcirculation, intravital microscopy demonstrated that the perfused boundary region, a marker of the barrier properties of endothelial glycocalyx, was higher in 40 SSc patients compared with 10 controls, suggestive of dysfunctional glycocalyx (79).

## Conclusions and therapeutic perspectives

**Figure 2:**
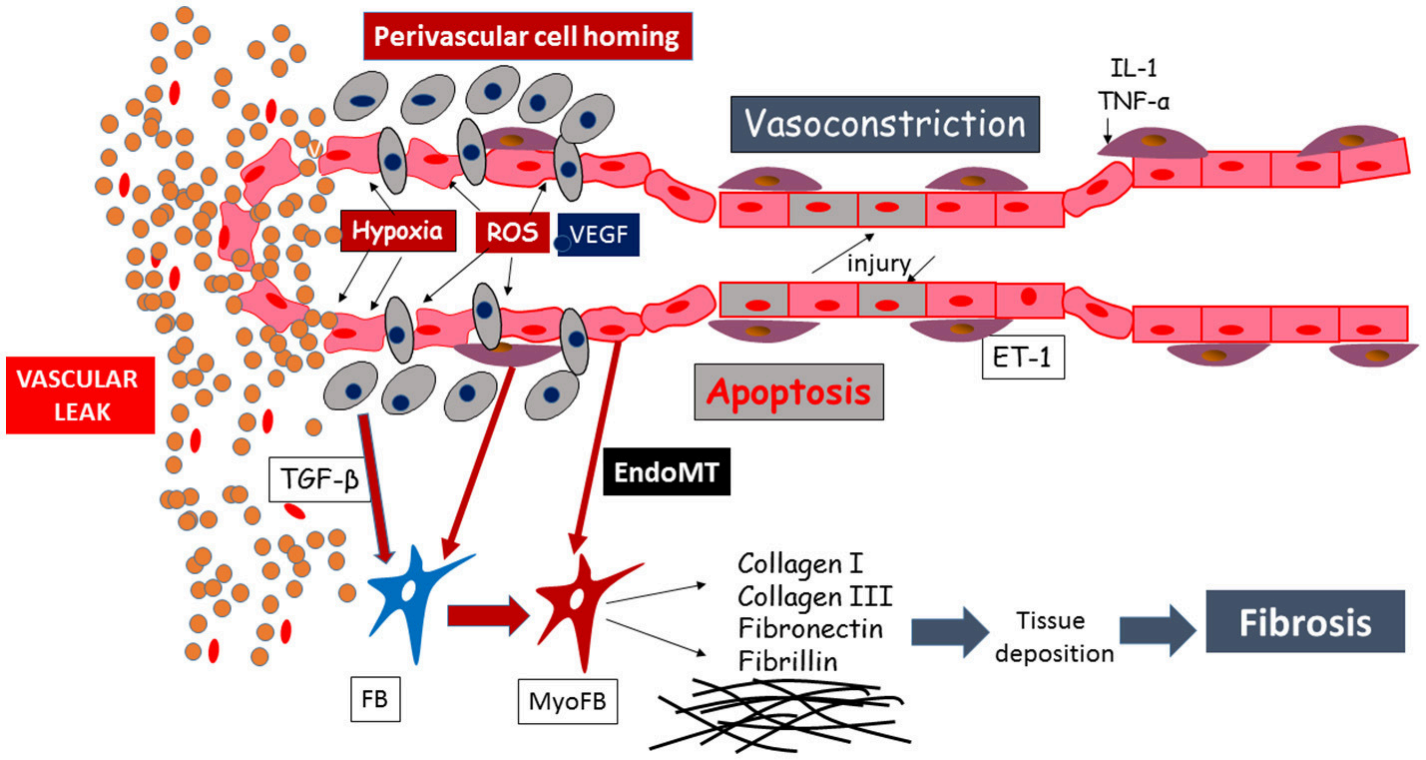
Schematic representation of the mechanisms leading to endothelial injury and capillary sufferance, evolving into vascular leaking.

Degradation of the endothelial barrier occurs in response to perivascular inflammation and to reactive oxygen species (ROS) generated by the ischemia-reperfusion injury, taking place in SSc patients' microvasculature (Figure 2). This phenomenon is a noxious trigger to the endothelial barrier leading to opening of the endothelial junctions, inflammatory cells homing, sustained hyperpermeability and continuous vascular leak (Figure 2). In early SSc, vascular and lymphatic angiopathy leads to leaking into tissue and interstitial edema generation, clinically visible as puffy fingers and detectable in NVC as a fluffy appearance of giant capillaries. It is clear that this phenomenon is at its zenith in the early edematous disease phase, is tightly linked to significant perivascular homing of inflammatory cells and progressively disappears with disease progression (32). From the pathogenetic point of view, vascular leaking testifies to the beginning of a process concentrated in the vessel wall involving ECs, inflammatory cells as well as the other tissue components. A pivotal role in these processes may be played by endothelin-1, which was shown to drive vascular fragility and endothelial dysfunction in animal models (80, 81), regulate adhesion molecules expression (82) and cellular migration (83), and to promote EndoMT (84). This was also indirectly proven by the amelioration induced by endothelin-1 receptor antagonists, manifested both clinically (85) and in NVC (86).

Therefore, the early SSc phase may be the ideal target to achieve the paradigm “to close the door,” i.e., prevent leaking into the tissues, mainly with a twofold strategy. The first to induce disease remission with immune suppression by blocking the vascular leaking, and in particular cellular trafficking, and therefore the progression of the disease. The second, to achieve vasoactive protection, restoring endothelial function and block remodeling of the vessel wall. This combination regimen may impact on disease evolution avoiding the progression to fibrosis. The induction therapy dealing with early vascular leaking needs to be carefully tested, including the possibility of using early intense immunosuppression followed by a lower dose “maintenance” treatment, assisted by vasoactive treatment and, in case of already manifested fibrotic changes, anti-fibrotic compounds (87).

In the future, increasing attention to vascular leaking is warranted to better understand early SSc and to evaluate a new strategic targeted therapy.

## Author contributions

CB, MM-C, and SB-R conceived the study and contributed to the draft of the manuscript. TF, MM, FWR, DEF, ADP, FR, and SG contributed to the draft of the manuscript.

### Conflict of interest statement

The authors declare that the research was conducted in the absence of any commercial or financial relationships that could be construed as a potential conflict of interest.
